# Adaptive Evolution Hotspots at the GC-Extremes of the Human Genome: Evidence for Two Functionally Distinct Pathways of Positive Selection

**DOI:** 10.1155/2010/856825

**Published:** 2010-05-03

**Authors:** Clara S. M. Tang, Richard J. Epstein

**Affiliations:** ^1^Laboratory of Computational Oncology, Department of Medicine, The University of Hong Kong, Pokfulam, Hong Kong; ^2^Department of Medicine, Queen Mary Hospital, University of Hong Kong, Pokfulam Rd, Pokfulam, Hong Kong

## Abstract

We recently reported that the human genome is ‘‘splitting” into two gene subgroups characterised by polarised GC content (Tang et al, 2007), and that such evolutionary change may be accelerated by programmed genetic instability (Zhao et al, 2008). Here we extend this work by mapping the presence of two separate high-evolutionary-rate (Ka/Ks) hotspots in the human genome—one characterized by low GC content, high intron length, and low gene expression, and the other by high GC content, high exon number, and high gene expression. This finding suggests that at least two different mechanisms mediate adaptive genetic evolution in higher organisms: (1) intron lengthening and reduced repair in hypermethylated lowly-transcribed genes, and (2) duplication and/or insertion events affecting highly-transcribed genes, creating low-essentiality satellite daughter genes in nearby regions of active chromatin. Since the latter mechanism is expected to be far more efficient than the former in generating variant genes that increase fitnesss, these results also provide a potential explanation for the controversial value of sequence analysis in defining positively selected genes.

## 1. Introduction

The genomes of higher species are under negative selection to maintain complexity, yet must also remain adaptable in order to defer extinction in changing environments. The genetic mechanisms that facilitate environmental adaptation, evolvability, and/or speciation in higher organisms remain unclear [2–5]; equally controversial are the criteria for defining and/or identifying positive selection, and for distinguishing adaptive evolution from neutral divergence and genetic drift [6–8]. Geographical isolation and inbreeding accelerate positive selection [9]—particularly for genes related to sexual pheromones, mate choice, fertility or neurodevelopment, many of which have been implicated by sequence (Ka/Ks) analysis [10–12]. Whether such analyses suffice for sensitive and specific detection of positively selected genes, however, is debated [13, 14]. 

 Positive selection does not occur randomly [15]. Relevant to this, we used methylation-sensitive dinucleotide and Ka/Ks analyses to show that promoter CpG islands act as evolutionary oscillators—that is, associated with increased transcription and low evolutionary rate when hypomethylated, but with low transcription and high evolutionary rate when hypermethylated [16]. Prior to this we reported a positive correlation between intron length and 3′ gene evolutionary rate, suggesting that this association reflected DNA misrepair due to intron-dependent transcriptional attrition [17]. In the present study, we have combined these experimental approaches to quantify the relative contributions of intron lengthening and methylation-dependent transcriptional silencing/mutation to gene evolutionary rates. Unexpectedly, the results implicate two separate pathways to adaptive evolution, at least one of which seems likely to involve gene duplication and/or exon insertion events affecting highly-transcribed, high-essentiality genes.

## 2. Materials and Methods

### 2.1. Sequence Data

We retrieved the genomic human sequence from the University of California, Santa Cruz (UCSC) Table Browser (http://genome.ucsc.edu/) [18]. Genome assemblies of hg18 (NCBI build 36.1, March 2006) were used. Sequence analyses were carried out using the entire data set of approximately 24,000 RefSeq genes, of which 15409 were informative. To prevent interspersed repeats like *Alu *sequences from creating bias in nucleotide composition, RepeatMask sequences were used. Genes not commencing with ATG codons, or not terminating with canonical stop codons, were excluded in order to obtain the most homogeneous set of coding genes. When several genes contained identical exonic sequences, only the one with the longest genomic length was retained. 

### 2.2. Distribution of GC Content

Distributions of coding GC % were best-fitted using the NOCOM program (http://www.genemapping.cn/nocom.htm) based on a counting (EM) algorithm. Under no transformation (exponent = 1), mean, the standard deviation and proportion of each population was estimated.

### 2.3. Gene Expression

The SAGEmap (Nov 2005, ftp://ftp.ncbi.nlm.nih.gov/pub/sage) of NCBI was used for quantitative evaluation of gene expression. SAGE libraries were grouped according to 26 tissue types including brain, blood, bone, bone marrow, cervix, cartilage, colon, eye, heart, kidney, liver, lung, lymph node, mammary gland, muscle, ovary, pancreas, peripheral nervous system, placenta, prostate, skin, stem cell, stomach, thyroid, vascular, and esophagus. Reliable tag-to-gene mapping of NlaIII SAGE tags to UniGene clusters was obtained from SAGEmap, and each cluster was represented by the longest RefSeq gene. Ambiguous tags mapping to more than one RefSeq gene were excluded. If a tag had been counted once only in one tissue, it was regarded as likely due to sequencing error and was thus discounted. SAGE tags of each RefGene were counted for each tissue type and normalized to counts per million. The normalized counts of each tissue were averaged across all tissue types for fair comparison between organs with different mean expression level.

### 2.4. Evolutionary Rate Determination

Homologue data in XML format was obtained from NCBI HomoloGene database (ftp://ftp.ncbi.nih.gov/pub/HomoloGene/). Orthologous gene pairs between human and mouse, together with their synonymous substitution, nonsynonymous substitution rate (Ka), and their ratio (Ka/Ks) were isolated.

## 3. Results

### 3.1. Two Separate GC-Content Peaks Are Demonstrable for Faster-Evolving Genes

**Figure 1:**
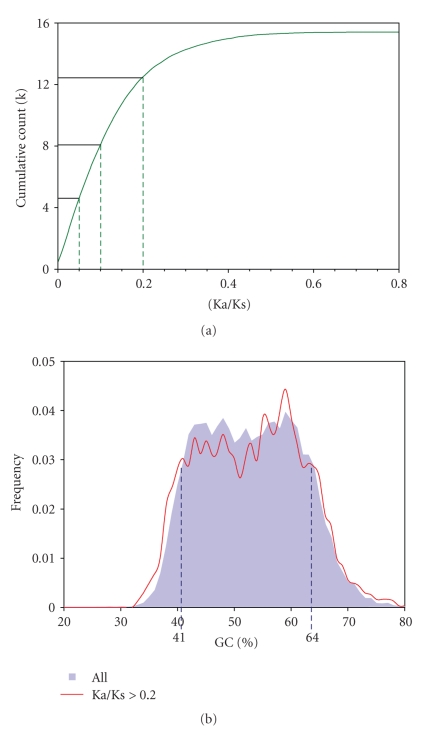
Ka/Ks profile of the human genome, showing that 75% of all genes are characterized by a Ka/Ks < 0.2; that is, most are under negative selection, whereas only a small percentage is characterised by very high Ka/Ks.

To explore the finding of an overall inverse trend between GC content and Ka/Ks noted in our last study [16], we first sought to determine the nature of this relationship using a specific gene set. To this end, we used the superfamily of human genes encoding G-protein-coupled receptors, including gene subsets encoding olfactory receptors, (putative) taste receptors, and putative vomeronasal receptors. Since many members of these gene families are believed to be transcriptionally inactive in humans, we expected a higher-than-usual proportion of high Ka/Ks (“pseudogenizing”) genes. Supplementary Figure 1(Supplementary Material available at doi:10.1155/2010/856825) suggests a negative relationship between GC content and Ka/Ks within this gene superfamily, consistent with an evolutionary role for methylation-dependent transcriptional inactivation and mutation. To extend our earlier finding of two GC-content gene modes within the human genome as a whole [16], we focused subsequent genomic analysis on a subset of genes with Ka/Ks > 0.2. This shows that most of these faster-evolving genes are characterized by GC contents less than 41%, with a relative scarcity of such genes in the 41–55% GC content range; but an additional fast-evolving gene subset is also detectable within the GC content range of 55–75% (Figure 1).

### 3.2. High-GC-Content Genes with Higher Ka/Ks Are Characterized by Relatively Higher Exon Numbers, Corrected for Gene Length, than Low-GC-Content High Ka/Ks Genes

**Figure 2:**
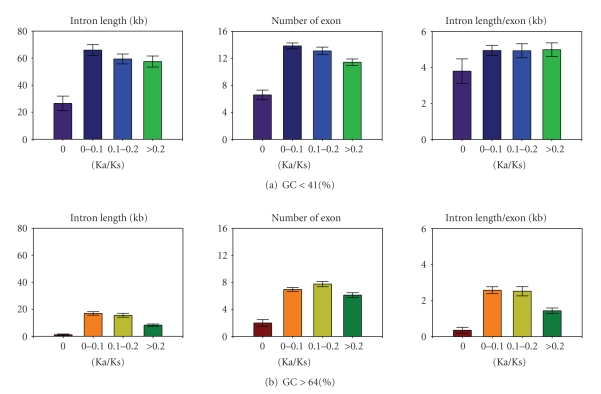
Comparative relationship between low- (upper rows) and high-GC gene groups (lower rows) and intron length (left) and exon number (middle), and their ratio (right).

The “golden middle” (highly regulated, intermediate-expressing genes) of the genome is reported to contain the longest genes [19], but this analysis has not been corrected for GC content. We find that subsets of rapidly evolving (Ka/Ks > 0.2) genes with low gene expression levels and breadth are identifiable within both low-GC (<41% GC content; *n* = 346) and high-GC (>64% GC content; *n* = 365) gene populations (*P* < 2.2 × 10^−16^, and *P* < .001, resp., Table 1). In contrast, more rapidly evolving high-GC genes exhibit an increase in exon number that is disproportionate to gene length, whereas low-GC genes do not (Figure 2). This difference raises the novel possibility that faster evolution of some high-GC genes could be mediated through exon insertion events, consistent with the notion that high-GC genes tend to be located within regions of accessible chromatin.

### 3.3. Both Low-GC and High-GC Ka/Ks Peaks Are Associated with Gene Lengthening as Transcription Declines

**Figure 3:**
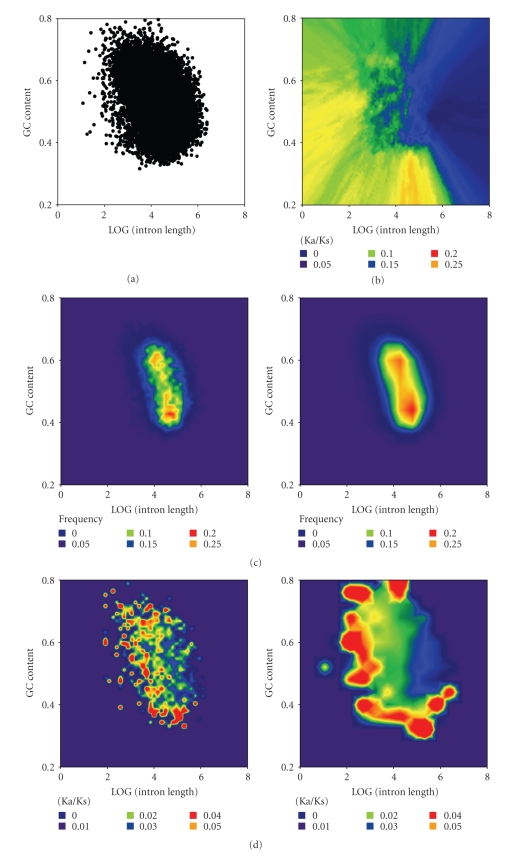

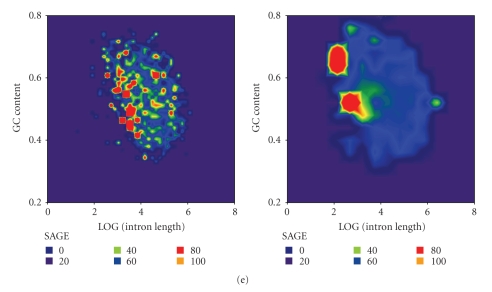
Distribution of genes with various GC content and intron length ((a), left) dot plot ((b), right) contour map with nearest neighbour smoothing. (c) Contour map with fixed neighbour smoothing (left, 1%) and (right, 5%). (d, e). Contour map of (d) Ka/Ks and (e) expression levels in SAGE of genes, using different sensitivity cutoffs (left, 1%, and right, 5%).

Three-dimensional genomic heat mapping was then used to characterise the foregoing Ka/Ks “twin peaks” in greater detail. Figure 3(a) confirms the negative relationship between GC content and gene length, while Figure 3(c) again suggests the existence of two discrete gene populations (a higher GC subgroup with shorter length, and a lower GC subgroup with higher length). The most transcribed genes tend to be those characterized by shorter gene length and intermediate-to-high GC content, with expression levels generally falling in association with longer gene/intron length (Figure 3(e)). Interestingly, genes with the highest Ka/Ks values are most obvious at lower GC and higher gene lengths (Figure 3(d), left panel), but at lower cutoffs are seen to track in a C-shaped distribution that overlies short, highly-transcribed genes and extends rightwards (i.e., in association with higher gene/intron lengths) when the two GC-extremes of the gene census are reached. Considered together with Table 1 and Figure 2, these data suggest that highly-transcribed genes (which, presumably, tend to be under strong negative selection) may give rise to less essential gene progeny via two different processes: either by gene methylation associated with reduced transcription, reduced repair of methylation damage (i.e., progressive CpG loss), and intron lengthening or by duplication and/or exon insertions affecting stably hypomethylated (high-GC) genes.

### 3.4. Gene Evolutionary Rate Tends to Be More Rapid in High-GC Genes with Higher Ratios of Exon Number to Intron Length

A weak-positive correlation exists between intron number and intron length, as expected, and two groups of outlier genes from the central distribution can be identified: shorter genes with relatively higher ratios of intron (exon) number to intron length and longer genes of relatively low exon:intron length ratio (Supplementary Figure 2). When compared using three-dimensional mapping, these latter two gene subsets are seen to differ in terms of gene expression levels and evolutionary rate, both of which appear higher in the shorter, high-exon group (Table 2; *P* < .03). The bimodality of high Ka/Ks genes when analysed in this way, independent of GC content, again suggests two distinct gene-altering pathways, one of which favors exon insertion over intron lengthening as a presumed adaptive mechanism.

## 4. Discussion

Biologydepends upon an environmentally-modulated balance between genetic conservation and variation [20–25]—implying, paradoxically, that genetic “variability” is somehow “conserved” at the species level so that fitness may be maintained. Evolutionary devices that may fulfil this need include introns and DNA methylation [26, 27]; by promoting both transcriptional inhibition and gene sequence mutation, the latter mechanism expedites rapid structural alterations of “underperforming” (i.e., less essential, pseudogenizing) genes [28]. The efficiency of such putative random mutations in producing selectable genes that confer a biological advantage can reasonably be predicted to be low [29], however, prompting the question whether more direct adaptive pathways to genetic novelty exist. 

 Relevant to this issue, horizontal gene transfer is increasingly recognized as a critical contributor to adaptive genomic evolution in prokaryotes [30]. In sexually reproducing organisms, analogous “horizontal” pathways to genomic change include not only retrotransposition, but also recombination, insertional mutagenesis (including exon swapping), and gene duplication/conversion or amplification [31]. The latter mechanism is attractive from a theoretical standpoint since prior conservation of an active gene per se implies functional conferral of a fitness advantage to a complex organism [32], thereby increasing the probability that a duplicated variant will offer further survival benefits [33, 34]. Consistent with this, human segmental duplications tend to occur around core duplicons which encode primate-specific genes under positive selection [35, 36]; similarly, duplications have been reported to be centred on positive selection hotspots for mating-specific genes [11]. Moreover, just as cellular stress has been shown to facilitate gene amplification [37, 38], it is tempting to postulate that transcriptional frequency and associated chromatin accessibility could directly promote adaptive gene duplication/conversion events [39]. 

 The findings of the present study are pertinent to the latter possibility. Our unexpected identification of a rapidly evolving human gene subgroup characterised by high GC content, relatively short gene length, but high ratio of exon number to intron length compared to slowly evolving genes of similar GC content, supports the view that positive selection may occur not only through passive release of negative selection constraints, but also via a more accelerated and direct mechanism involving, say, exon insertion into GC-rich duplicates of ancestral genes characterized by high expression and tight conservation. Of note, this putative pathway of positive selection is quantitatively underestimated by studies based on point mutation (Ka/Ks) data alone, since most of the functional novelty is predicted to arise either from changes in chromosomal gene location affecting expression [39] or from exon insertion events unassociated with sequence variation. Indeed, recent work from Drummond and Wilke [40] suggests that protein misfolding may be the dominant selection pressure in metazoan evolution, casting further doubt on the equation of Ka/Ks with evolutionary rate. Interestingly, Jordan et alhave shown that gene essentiality selectively correlates with evolutionary conservation in bacterial genomes, though not in mammalian [41]. These and other reports emphasise that evolutionary rate is likely influenced by many complex and heterogeneous factors.

 The conclusions of our study remain limited by their inferential and non-specific nature. More direct evidence of positive selection based on experimental manipulation of gene duplication and related processes (conversion, amplification, recombination) is needed before any firm conclusions are drawn. Nonetheless, the prospect of accelerating species evolution by using global genomic techniques to promote gene duplication, even if only on an experimental basis initially, is exciting. Conversely, the possibility that maladaptive somatic processes such as cancer may be driven in part by positive selection secondary to such global genomic changes [42] is important to consider. Chromatin-based therapeutic interventions, either at the cellular (germline) or tissue (somatic) level, could be the long-term deliverable from this line of evolutionary investigation.

##  Conflict of Interest 

There is no conflict of interest.

##  Authors' Contributions 

Clara S. M. Tang performed the calculations and experiments, and helped finalize the manuscript. Richard J. Epstein designed the experiments and wrote the paper.

## Supplementary Material

Inverse relationship between GC content and Ka/Ks. Genes encoding olfactory receptors (golden triangles), which are known to undergo positive selection, and structurally related taste receptors–including vomeronasal receptors, which are non-functional in humans–were used to exemplify the relationship within a data set which is expected to be enriched for pseudogenization.

## Figures and Tables

**Table 1:** Mean expression score (breadth and SAGE) of varying Ka/Ks groups for low and high GC genes. The data confirm that the different Ka/Ks groups so defined vary significantly in terms of gene expression levels for both low-GC (correlation coefficient −0.32, *P* < 2.2 × 10^−16^) and high-GC gene subsets (correlation coefficient −0.10, *P* = .00033), as well as in terms of expression breadth (correlation coefficient −0.35, *P* < 2.2 × 10^−16^, and correlation coefficient −0.098, *P* = .00067, resp.) using Spearman correlation.

Ka/Ks	Breadth		SAGE	
	Low GC	High GC	Low GC	High GC
0	15.85	11.83	163.93	103.38
0–0.1	14.03	10.74	58.22	67.84
0.1–0.2	11.52	10.40	39.42	74.04
>0.2	9.10	8.86	32.37	57.75

**Table 2:** Characterisation of gene subsets with differing intron/exon numbers and intron length, in terms of evolutionary rate and gene expression. Spearman correlation coefficient (= 0.58, *P* < 2.0 × 10^−16^) was calculated for gene subgroups greater than 2SD (intron length/number and intron number/length) from the mean.

	Short and higher intron		Long and higher intron		*P-*value^†^
	length/number		number/length		
	Mean	Median	Mean	Median	
Ka/Ks	0.19	0.17	0.054	0.080	<2 × 10^−16^
Breadth	10.27	9	23	11.52	0.019
SAGE	114.44	34.66	30.05	39.91	0.021

^†^ *P-*value of nonparametric Mann-Whitney test.
