# Supplementary figures and images for: Adaptive Evolution Hotspots at the GC-Extremes of the Human Genome: Evidence for Two Functionally Distinct Pathways of Positive Selection
Source: Adv Bioinformatics. 2010 May 3;2010:856825. doi: 10.1155/2010/856825 (PMC2862947; doi:10.1155/2010/856825)

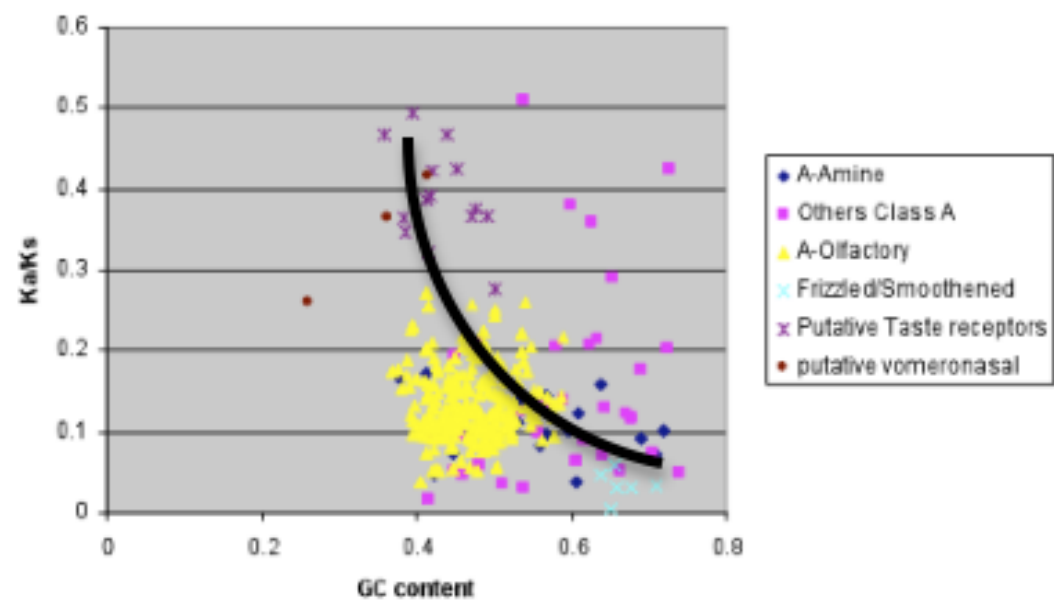

**Supplementary Figure 1**

Supplement: Supplementary file 1 — Inverse relationship between GC content and Ka/Ks. Genes encoding olfactory receptors (golden triangles), which are known to undergo positive selection, and structurally related taste receptors–including vomeronasal receptors, which are non-functional in humans–were used to exemplify the relationship within a data set which is expected to be enriched for pseudogenization. [file 856825.f1.pdf]
